# Combination of anxiety and depression is associated with an increased headache frequency in migraineurs: a population-based study

**DOI:** 10.1186/s12883-014-0238-4

**Published:** 2014-12-14

**Authors:** Kyungmi Oh, Soo-Jin Cho, Yun Kyung Chung, Jae-Moon Kim, Min Kyung Chu

**Affiliations:** Department of Neurology, Korea University Guro Hospital, Korea University School of Medicine, Seoul, Korea; Department of Neurology, Dongtan Sacred Heart Hospital, Hallym University College of Medicine, Hwaseong, Korea; Department of Occupational and Environmental Medicine, Sacred Heart Hospital, Hallym University College of Medicine, Anyang, Korea; Department of Neurology, Chungnam National University, College of Medicine, Daejeon, Korea; Department of Neurology, Sacred Heart Hospital, Hallym University College of Medicine, Anyang, Korea

**Keywords:** Migraine, Anxiety, Depression, Epidemiology, Comorbidity

## Abstract

**Background:**

Although anxiety and depression have been classified as distinct traits of affective disorders, previous studies have reported their co-occurrence in subjects with migraine. However, few reports are available on the clinical implications of this comorbidity. This study is to assess the comorbidity of anxiety and depression in subjects with migraine and its clinical implications in a population-based sample from Korea.

**Methods:**

We selected Korean subjects aged 19–69 years by the stratified random sampling method, and evaluated them using a semi-structured interview, designed to identify headache type, anxiety, and depression. We used Goldberg Anxiety Scale questions and Patient Health Questionnnaire-9 for the diagnosis of anxiety and depression, respectively.

**Results:**

Of the 2,762 participants who completed the interview, 147 subjects (5.4%) were classified as having a migraine during the previous year. Among these 147 subjects, 17 (11.6%) had anxiety and depression, 28 (19.0%) had anxiety alone, 9 (6.1%) had depression alone, and 93 (63.3%) had neither anxiety nor depression. Headache frequency per month was remarkably higher in subjects having migraine with anxiety and depression (median [25–75 percentile values], 8.0 [2.5–21.0]) than in those having migraine with anxiety alone (2.0 [1.0–5.0], *p* = 0.003), migraine with depression alone (1.0 [0.3–4.0], *p* = 0.001), and migraine without anxiety or depression (1.0 [0.3–3.0], *p* < 0.001). The migraine with anxiety alone (7.0 [6.0–8.0], *p* = 0.011) group and migraine with anxiety and depression (7.0 [5.0–9.0], *p* = 0.018) group showed higher Visual Analogue Scale scores for pain intensity compare to migraine without anxiety or depression (6.0 [5.0-7.0]) group.

**Conclusions:**

Approximately 1/3 of migraineurs with anxiety had depression and 2/3 of migraineurs with depression had anxiety. Combination of anxiety and depression was associated with an increased headache frequency. Anxiety was associated with exacerbation of headache intensity.

**Electronic supplementary material:**

The online version of this article (doi:10.1186/s12883-014-0238-4) contains supplementary material, which is available to authorized users.

## Background

Migraine is a common, disabling neurological disorder and its prevalence ranges between 5% and 12% in the general population [1-3]. The symptoms of migraine and associated psychiatric disturbances may cause disabilities that interfere with performance in educational and occupational pursuits and household chores [2-4]. The World Health Organization has recognized migraine as an urgent public health priority and listed it as the 7^th^ leading cause of disability [3].

Epidemiological and clinical studies have shown that migraine is comorbid with a number of psychiatric disorders such as anxiety and depression [5-9]. Headache frequency and impact of headache are more pronounced in migraine with comorbid psychiatric disorders than in migraine without comorbid psychiatric disorders [4,6]. Psychiatric comorbidities are more prevalent in chronic migraine (CM) compared to episodic migraine [10,11].

Anxiety is the most common psychiatric comorbidity among patients with migraine, showing a prevalence of 25.5–57.6% in population-based studies [5-7,10,12,13]. The Diagnostic and Statistical Manual of Mental Disorders-4 text revision (DSM-IV TR) identified anxiety as an Axis-1 disorder that represented acute symptoms requiring treatment [14]. Patients having migraine with anxiety are more likely to experience disability and poor quality of life compared to those without anxiety [6,9,13,15].

Depression is another Axis-1 disorder in the DSM-IV TR [14]. A close association between depression and migraine has been reported in clinical settings and population-based studies [7-10,12,13,16-18]. Depression may exacerbate the impact of migraine and complicate treatment [6,16]. Longitudinal studies have reported bidirectional comorbidity in which migraine predisposes patients to depression and vice versa [16,19-21]. A recent study has shown that depression is associated with the transformation from episodic to CM [22].

Although anxiety and depression have been classified as distinct traits of affective disorders, concurrence of anxiety and depression has been observed in clinical and epidemiological studies [13,23,24]. Most individuals with anxiety or depression possess these traits in mixed form, rather than having pure anxiety or pure depression. The concurrence of anxiety and depression in migraine has been reported; 42.1–84.6% of patients having migraine with depression also experience anxiety, and 66.1–85.7% of patients having migraine with anxiety also have depression [6-9,12,20].

However, the comorbidity of anxiety and depression in migraineurs and its clinical significance has rarely been reported. In the present study, we will: 1) describe the prevalence of anxiety, depression, and migraine in a general Korean population; 2) assess the comorbidity of anxiety and depression among subjects with migraine; 3) examine the clinical characteristics of subjects having migraine according to the diagnosis of anxiety and depression.

## Methods

This study provides a nation-wide, cross-sectional survey of headache and anxiety in the Korean population. Trained interviewers conducted structured interviews, using a questionnaire to diagnose headache disorders and anxiety in adults aged 19–69 years. The interview included questions on the symptoms and impact of headache and anxiety. Further, the socioeconomic, demographic, and geographic characteristics of participants were evaluated. This study was undertaken from November 2011 to January 2012, and was approved by the institutional review board/ethics committee of Hallym University Sacred Heart Hospital (Additional files 1 and 2). Written informed consent was obtained from all participants.

### Target area

Korea is geographically partitioned into 15 administrative divisions (“do”), except Jeju-do, and each administrative division is further divided into “si,” “gun,” or “gu” as the basic administrative units. In total, there are 77 si, 88 gun, and 69 gu. The estimated population of Korea in 2010 was 48,580,293, of which approximately 32,356,747 people were aged 19–69 years, as per data from the 2010 population and housing census by the National Statistical Office [25]. This study included all Korean territories except Jeju-do. We classified 7 metropolitan “si” areas (Seoul, Busan, Daegu, Incheon, Gwangju, Daejeon, and Ulsan) as “large cities,” other “si” areas as “medium-to-small cities,” and “gun” areas as “countryside” for this analysis.

### Sampling method

To determine the prevalence rates of and analyze the demographic factors affecting common primary headache disorders, we planned to sample 2,750 individuals based on the population structure. We adopted a 2-stage systematic random sampling method. The 15 administrative divisions were designated as the primary sampling unit. We assigned appropriate sample numbers for each primary sampling unit according to the population distribution. In the second stage, we further selected representative basic administrative units (si, gun, and gu) for each primary sampling unit. Overall, 60 representative basic administrative units were selected for this study. For each representative basic administrative unit, we assigned a target sample number regarding age, gender, and occupation. Estimated sampling error of our study is ±1.8% with a 95% confidence interval (Table 1) [26].

**Table 1:** **Sociodemographic distribution of all survey participants, the total Korean population, and of cases identified as migraine, anxiety and depression**

		**Sample number, N (%)**	**Total population, N (%)**	***p*** **-value**	**Migraine, N, % (95% CI)**	**Anxiety, N, %, (95% CI)**	**Depression, N, %, (95% CI)**
Gender							
	Men	1377 (49.2^a^)	16,357,919 (50.6)	0.42^b^	36, 2.6 (1.8-3.5^a^)	111, 8.1 (6.6-9.5^a^)	47, 3.4 (2.5-4.4^a^)
	Women	1385 (50.8^a^)	15,998,828 (49.4)		111, 8.0 (6.6-9.4^a^)	163, 11.8 (10.1-13.5^a^)	77, 5.6 (4.4-6.8^a^)
Age							
	19-29	542 (20.0^a^)	16,357,919 (50.6)	0.76^b^	25, 4.5 (2.8-6.2^a^)	53, 9.6 (7.2-12.1^a^)	23, 4.2 (2.5-5.8^a^)
	30-39	604 (21.3^a^)	15,998,828 (49.4)		42, 7.0 (4.9-9.1^a^)	51, 8.7 (6.4-11.0^a^)	32, 5.4 (3.6-7.3^a^)
	40-49	611 (22.5^a^)	16,357,919 (50.6)		39, 6.5 (4.5-8.4^a^)	67, 11.0 (8.5-13.5^a^)	24, 4.0 (2.5-5.5^a^)
	50-59	529 (18.4^a^)	15,998,828 (49.4)		22, 4.1 (2.4-5.9^a^)	53, 10.0 (7.3-12.5^a^)	22, 4.2 (2.5-6.0^a^)
	60-69	476 (17.8^a^)	16,357,919 (50.6)		19, 4.2 (2.4-6.0^a^)	50, 10.6 (7.8-13.3^a^)	23, 4.9 (2.9-6.8^a^)
Size of residential area							
	Large city	542 (20.0^a^)	1,5606,652 (48.2)	0.90^b^	76, 6.0 (4.7-7.3^a^)	130, 10.1 (8.5-11.8^a^)	59, 4.6 (3.5-5.8^a^)
	Medium-to-small city	604 (21.3^a^)	1,4106,687 (43.6)		50, 4.2 (3.0-5.2^a^)	115, 9.6 (7.9-11.2^a^)	50, 4.2 (3.1-5.3^a^)
	Rural area	611 (22.5^a^)	264,307 (8.2)		21, 7.8 (4.6-11.0^a^)	29, 10.6 (7.0-14.3^a^)	15, 5.6 (2.9-8.4^a^)
Educational level							
	Middle school or less	446 (16.5^a^)	6,147,782 (19.0)	0.90^b^	26, 5.8 (3.6-7.9^a^)	58, 13.0 (9.9-16.0^a^)	24, 5.5 (3.4-7.6^a^)
	High school	1218 (43.8^a^)	14,172,255 (43.8)		60, 5.0 (3.8-6.2^a^)	113, 9.3 (7.6-10.9^a^)	52, 4.3 (3.2-5.4^a^)
	College or more	1005 (38.7^a^)	1,2036,710 (37.2)		60, 5.6 (4.2-7.0^a^)	101, 9.5 (7.7-11.3^a^)	48, 4.5 (3.3-5.8^a^)
Total		2,762 (100.0^a^)	32,356,747 (100.0)		147, 5.4 (4.5-6.2^a^)	147, 5.4 (4.5-6.2^a^)	274, 10.0 (8.8-11.1^a^)

^a^Adjusted after age, gender, size of residential area and educational level.

^b^Compared gender, age group, size of residential area, and educational level distributions between the sample of the present study and total population of Korea.

### Survey procedures

Subjects were stratified according to age, gender, and occupation. Prior to meeting the subjects, the interviewers were provided with the following information: 1) the aims of the present study, 2) the meaning of each question, 3) instructions for interpreting the subjects’ responses, and 4) other details that were relevant to conducting a proper interview. All interviewers were employed by Gallup Korea and had previous socials survey interviewing experience. The interviewers were not medical personnel. The survey was conducted by door-to-door visits and face-to-face interviews.

### Diagnosis of migraine, anxiety, and depression

We diagnosed migraine, anxiety, and depression using a questionnaire composed of three parts (Additional files 3 and 4). The first part assessed demographic and socioeconomic characteristics. The second part established a headache profile, which was designed to comply with ICHD-2. Migraine was diagnosed based on ICHD-2 criteria for migraine without aura [27]. We did not attempt to diagnose migraine with aura and migraine without aura separately and classified them as migraine in the present study. The questions used to diagnose migraine were previously found to have 75.0% sensitivity and 88.2% specificity, by comparing the diagnoses from the survey with doctors’ diagnoses obtained from an additional telephone interview [28].

The third part included questions about anxiety and depression. We used the Goldberg Anxiety Scale (GAS) for diagnosing anxiety. GAS is composed of four screening questions and five supplementary questions [29,30]. If a participant gave a positive answer for two or more of the first four screening questions, and five or more of all GAS questions, he/she was diagnosed with anxiety. The Korean version of GAS was reported to have 82.0% sensitivity and 94.4% specificity [30]. The Korean version GAS showed a good correlations with State-Trait Anxiety Inventory, a validated tools for assessing anxiety [31,32].

The Patient Health Questionnaire-9 (PHQ-9) was used for diagnosing depression [33]. If a participant’s PHQ-9 score was 10 or more, he/she was assigned as having depression. The Korean PHQ-9 was found to have 81.1% sensitivity and 89.9% specificity [34]. We included the Headache Impact Test-6 questionnaire (HIT-6) to evaluate the impact of headache on quality of life.

### Analyses

Based on the definitions of migraine and anxiety, the 1-year prevalence was presented as the number of cases per 100 persons. Age- and gender-specific prevalence was also calculated. The Kolmogorov–Smirnov test was used to test for normality of the distribution.

We calculated the odds ratios (OR; 95% confidence intervals [CI]) for occurrence anxiety or depression with migraine compared to that without migraine using univariate and multivariate logistic regression analyses. In univariable analyses, we modeled the ORs for migraine versus non-migraine without adjusting for covariates. In multivariable-adjusted analyses for anxiety, sociodemographic variables (age, gender, educational level, and size of residential area) and depression were used as covariates. In multivariate analyses for depression, sociodemographic variables and anxiety were used as covariates.

We classified subjects with migraine into four groups according to their diagnoses for anxiety and depression: the migraine without anxiety or depression, migraine with anxiety alone, migraine with depression alone, and migraine with anxiety and depression. We compared headache days per month, visual analogue scale (VAS) score of headache intensity, and HIT-6 score, among these four groups using the Kruskal–Wallis one-way analysis of variance test. If the median values were significantly different, post-hoc tests were carried out using Bonferroni’s method. In all statistical analyses, the significance level was 0.05, unless otherwise specified. The results were analyzed using the Statistical Package for the Social Sciences 21.0 (SPSS 21.0; IBM, Armonk, NY, USA).

As with most survey sampling designs, missing data resulting from non-response occurred in several variables. The data reported are based on the available data. Sample sizes of some variables diverge from the sample size of n = 2,762 because of non-responses on that particular variable. Imputation techniques were not employed to minimize non-response effects [35].

## Results

**Figure 1:**
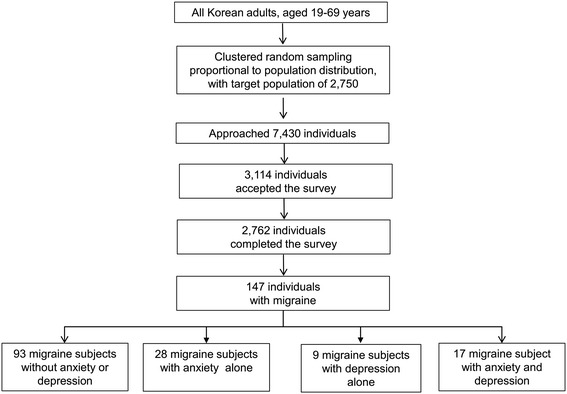
**Flow chart depicting the participation of the Korean Headache-Sleep study.**

Our interviewers approached 7,430 individuals and 3,114 of them accepted the survey (rejection rate of 58.1%). After 352 individuals suspended the interview, 2,762 subjects completed the survey (cooperation rate of 37.2%; Figure 1). Distributions of age, gender, size of residential area, and educational level were not significantly different from those of the general population of Korea (Figure 1 and Table 1).

### Prevalence of migraine, anxiety, and depression

Of the 2,762 participants, 1,299 (47.2%) subjects had had at least one attack of headache, and 147 subjects (5.4%) were classified as having migraine during the previous year, 274 (10.0%) were classified as having anxiety, and 124 (4.5%) were classified as having depression (Table 1).

### Anxiety and depression in subjects with migraine

**Figure 2:**
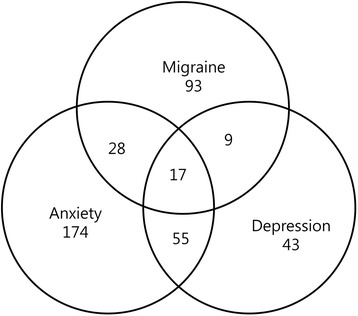
**Venn diagram for distribution of subjects with anxiety, depression and migraine.**

There was considerable overlap between anxiety and depression among subjects with migraine. Of the 147 subjects with migraine, 28 (19.0%) had anxiety alone, 9 (6.1%) had depression alone, and 17 (11.6%) had anxiety and depression. The remaining 93 (63.3%) had neither anxiety nor depression (Figure 2). The prevalence of anxiety in subjects with migraine (30.1%) was higher than that in subjects without migraine (8.8%, OR = 4.5, 95% CI 3.1–6.5); this pattern was consistent even after adjusting for sociodemographic variables (age, gender, educational level, and size of residential area) and depression (OR = 3.0, 95% CI = 2.0-4.7). The prevalence of depression in subjects with migraine (17.7%) was higher than that in subjects without migraine (3.8%, OR = 5.4, 95% CI 3.4–8.7); this pattern was consistent even after adjusting for sociodemographic variables and anxiety (OR = 2.7, 95% CI = 1.6-4.7).

### Clinical characteristics of subjects having migraine with and without anxiety and depression

We investigated demographics, headache characteristics, associated symptoms, headache frequency per month, VAS score for pain intensity, and HIT-6 score of subjects with migraine grouped according to presence of anxiety and depression. Demographic distributions, headache characteristics and associated symptoms of migraineurs were not significantly different according to anxiety or depression except photophobia (Table 2). Photophobia was less prevalent in migraineurs with depression alone compared to migraineurs without anxiety or depression (*p* = 0.007), migraineurs with anxiety alone (*p* = 0.001), and migraineurs with anxiety and depression (*p* = 0.012).

**Table 2:** **Demographics, headache characteristics and associated symptoms of migraineurs according to anxiety and depression**

		**Migraine subjects without anxiety or depression, N = 93**	**Migraine subjects with anxiety alone, N = 28**	**Migraine subjects with depression alone, N = 9**	**Migraine with anxiety and depression, N = 17**	**P-value**
Demographics						
	Mean age ± SD (years)	40.4 ± 11.4	47.2 ± 14.5	42.1 ± 15.7	44.5 ± 18.4	0.131
	Women, N (%)	71 (75.5)	21 (77.8)	7 (77.8)	13 (76.5)	0.995
Headache characteristics						
	Unilateral pain, N (%)	57 (60.6)	15 (53.6)	4 (44.4)	7 (41.2)	0.409
	Pulsating quality, N (%)	72 (76.6)	21 (77.8)	7 (77.8)	12 (70.6)	0.950
	Moderate-to-severe severity, N (%)	72 (75.8)	22 (81.5)	8 (88.9)	17 (100.0)	0.119
	Aggravation by movement, N (%)	60 (63.8)	21 (77.8)	5 (55.6)	15 (88.2)	0.121
Associated symptoms						
	Nausea, N (%)	84 (88.4)	23 (82.1)	9 (100.0)	15 (88.2)	0.544
	Vomiting, N (%)	33 (34.7)	15 (53.6)	3 (33.3)	8 (47.1)	0.288
	Photophobia, N (%)	55 (57.9)	19 (70.4)	1 (11.1)	11 (64.7)	0.017
	Phonophobia, N (%)	63 (67.0)	21 (77.8)	6 (66.7)	13 (76.5)	0.668
	Osmophobia, N (%)	42 (44.7)	13 (48.1)	4 (44.4)	9 (46.3)	0.930

Headache frequency was remarkably higher in the migraine with anxiety and depression group (median [25–75 percentile values], 8.0 [2.5–21.0]) compared to that in the migraine with anxiety alone (2.0 [1.0–5.0], *p* = 0.003), migraine with depression alone (1.0 [0.3–4.0], *p* = 0.001), and migraine without anxiety or depression (1.0 [0.3–3.0], *p* < 0.001) groups (Table 3). The migraine with anxiety alone group showed higher VAS scores for pain intensity compared to the migraine without anxiety or depression group (7.0 [6.0–8.0] vs. 6.0 [5.0–7.0], *p* = 0.011). The VAS score of the migraine with anxiety and depression group was not significantly different from that of the migraine with anxiety alone group (7.0 [5.0–9.0] vs. 7.0 [6.0–8.0], *p* = 1.000). The HIT-6 score was higher for the migraine with depression alone group than for the migraine without anxiety or depression group (50.0 [46.0–58.0] vs. 62.0 [52.0–70.5], *p* = 0.004). This score did not significantly differ between the migraine with anxiety and depression and migraine with depression alone groups (64.0 [61.0–67.0] vs. 62.0 [52.0–70.5], *p* = 1.000).

**Table 3:** **Frequency, severity and impact of headache according to migraineurs’ anxiety and depression status**

	**Migraine subjects without anxiety or depression, N = 93 Median (25% -75%)**	**Migraine subjects with anxiety alone, N = 28 median (25% -75%)**	**Migraine subjects with depression alone, N = 9 median (25% -75%)**	**Migraine subjects with anxiety and depression, N = 17 median (25% -75%)**	P-value*	**Post hoc analysis with Bonferroni’s correction**
Frequency per month	1.0 (0.3-3.0)	2.0 (1.0-5.0)	1.0 (0.3-4.0)	8.0 (2.5-21.0)	<0.001	1 vs. 2 = 0.596
						1 vs.3 = 1.000
						1 vs. 4 < 0.001
						2 vs.3 = 1.000
						2 vs. 4 = 0.003
						3 vs. 4 = 0.001
VAS score for pain intensity	6.0 (5.0-7.0)	7.0 (6.0-8.0)	7.0 (6.0-8.0)	7.0 (5.0-9.0)	<0.001	1 vs. 2 = 0.011
						1 vs.3 = 0.824
						1 vs. 4 = 0.018
						2 vs.3 = 1.000
						2 vs. 4 = 1.000
						3 vs. 4 = 1.000
HIT-6 score	50.0 (46.0-58.0)	57.0 (49.0-60.8)	62.0 (52.0-70.5)	64.0 (61.0-67.0)	<0.001	1 vs. 2 = 0.074
						1 vs.3 = 0.004
						1 vs. 4 < 0.001
						2 vs.3 = 0.545
						2 vs. 4 = 0.005
						3 vs. 4 = 1.000

HIT-6: Headache Impact Test-6; VAS: Visual Analogue Scale.

*Kruskal–Wallis one-way analysis of variance test among the four groups: migraine subjects without anxiety or depression, migraine subjects with anxiety alone, migraine subjects with depression alone and migraine subjects with anxiety and depression.

1: migraine subjects without anxiety or depression; 2: migraine subjects with anxiety alone; 3: migraine subjects with depression alone; 4: migraine with anxiety and depression.

## Discussion

The key findings of the present study are as follows: 1) The prevalence of anxiety, depression, and migraine in the Korean population were 10.0%, 4.5% and 5.4%, respectively; 2) Among subjects with migraine, 19.0% had anxiety alone, 6.1% had depression alone, and 11.6% had both anxiety and depression; 3) Headache frequency was markedly increased in subjects having migraine with anxiety and depression.

The 1-year migraine prevalence rate (5.4%) in the present study was somewhat lower than those previously done in European (10-25%) and North American (9-16%) countries [2]. However, the migraine prevalence rate in the present study was similar to those of previous studies in Korea and Asian countries [28,36]. The 1-year prevalence rate of migraine in Asian countries mostly ranged from 4.7% to 9.1%, which were somewhat lower than those of European and North American countries [36].

The prevalence of anxiety and depression in this study were similar to those previously reported, ranging from 5.6% to 19.3% for anxiety [37-39] and 3.6% to 8.8% for depression [40-42]. This indicates the reliability of the present study. The wide range of prevalence values reported in previous studies may be explained by differences in ethnicity, cultural background, survey methods, and assessment tools.

Previous studies have reported an association between migraine and psychiatric comorbidities such as anxiety and depression [5-7]. The clinical relationship between migraine and psychiatric comorbidities implies the need for judicious evaluation of a patient with migraine for presence of other conditions. In addition, for patients having comorbid conditions, treatment choices that can improve both conditions should be considered [43].

Numerous studies have shown a close association between depression and migraine. As per population-based studies, subjects with migraine are 2.2–3.5 times more likely to develop depressive disorders than those without migraine [5,7,21,44]. Anxiety has also been reported to show a significant association with migraine, and subjects with migraine have increased risk of anxiety, with ORs of 2.3–2.8 [6,7,9,16]. In the present study, subjects with migraine showed increased risk of anxiety and depression, with ORs of 3.0 and 2.7, respectively.

Previous epidemiological and clinical studies have reported that anxiety and depression commonly co-occur in individuals with migraine [5-7]. Breslau et al. reported that depression and anxiety co-exist in approximately 30% of patients with migraine. In their population-based study, 84% of patients having migraine with depression also had anxiety, and 54% of patients having migraine with anxiety had depression [45]. Another study from France reported that 84.6% of patients having migraine with depression had anxiety and 40.4% of patients having migraine with anxiety had depression [6]. In the current study, 11.5% (17/147) of subjects with migraine had anxiety and depression; among subjects having migraine with depression, 65.4% (17/26) had anxiety, and among subjects having migraine with anxiety, 37.8% (17/45) had depression. The discrepancy between the percentages reported in the present and previous studies may be partially explained by differences in ethnicity, cultural background, and assessment methods.

The negative effect of anxiety and depression on headache-related disability and the impact of headache on quality of life have been documented [6,9,15,18]. Depression is related to disease prognosis, transformation to CM, and treatment outcomes [7-10,12,13,17,22]. Patients having migraine with anxiety show higher levels of migraine-related disability [6,11]. In migraine with concurrent anxiety and depression, the migraine-related disability extends beyond that associated with comorbid anxiety alone [6]. However, the associations between anxiety or depression and clinical characteristics of migraine are currently less well known.

In the current study, we investigated the relation of headache frequency, headache severity, and impact of headache with the presence of anxiety and depression (Table 3). Headache frequency of subjects having migraine with either anxiety or depression alone was not significantly different from that of subjects having migraine without anxiety or depression. However, in subjects having migraine with anxiety and depression, headache frequency was remarkably higher. Since headache frequency is an important factor for migraine chronification, our finding suggests that concurrent anxiety and depression may be associated with migraine chronification. Further longitudinal studies examining the role of anxiety and depression in migraine chronification would provide a better understanding of the psychological factors involved in migraine. The VAS score for pain intensity showed a different pattern from that of headache frequency. The score was elevated when anxiety was present, and the VAS score for migraine with anxiety and depression was not significantly different from that for migraine with anxiety alone. The association between anxiety and elevation of VAS score may be partially explained by a psychological component of pain perception [46].

Although the response rate is not high, we used 2-stage clustered random sampling pool of Gallup Korea, which showed a low sampling error [47]. The socio-demographic distributions of participants were similar to those of total population of Korea (Table 1). The prevalence rates of migraine, anxiety and depression in our survey were similar to those studies previously done in Korea [26,28,42]. Use of reliable sampling method, similarity in socio-demographic distributions to whole population of Korea and similarity in migraine, anxiety and depression prevalence rate to previous studies suggested that our study properly reflected migraine status of Korean population.

In this study, we did not attempt to examine the presence of aura because the diagnosis of aura is difficult to verify using questionnaire [48]. According to the ICHD-2 criteria, migraine with aura was diagnosed when an individual’s headache fulfils typical headache features and presence of aura [27]. Typical headache features of migraine with aura was the same as those of migraine without aura. We investigated typical headache features of migraine without aura for the diagnosis of migraine and included both migraine without aura and migraine with aura as migraine in the present study.

We did not try to diagnose CM in the present study because the diagnostic criteria for CM were currently under amendment and investigating CM in epidemiological study was very difficult [49-52]. The most recent criteria, the third beta edition of International Classification of Headache Disorder, define CM as a headache on ≥15 days per month, including a migraine with/without an aura or relieved by a triptan or ergot derivatives on at least 8 days per month, with a duration of at least 3 months and are difficult to apply in epidemiological study investigating CM using questionnaire [51]. Further studies regarding comorbidity of anxiety and depression in CM sufferers and their clinical significance in CM would be needed.

This study has some limitations. Firstly, we used GAS to diagnose anxiety. GAS is composed of nine questions on symptoms of anxiety, and one question about a participant’s headaches or neck aches. Based on these questions, subjects with migraine may have obtained higher GAS scores, and hence, be more likely to be diagnosed as having anxiety. However, we think that our results for comorbidity of anxiety and migraine are reliable because of the following reasons: 1) The usefulness of the Korean version GAS for anxiety diagnosis has been validated, with the Korean GAS showing high sensitivity and specificity; [30] 2) Subjects with migraine showed a higher prevalence of anxiety compared to those with non-migraine headaches, who might have also responded positively to the question on head and neck aches (13.3% vs. 30.1%, *p* < 0.001); 3) We observed a quantitative relationship between migraine clinical characteristics and anxiety, and a higher VAS score in migraine with anxiety. Secondly, although this is a population-based study with a low sampling error, its statistical power for examining subgroups was limited. Thus, some results might have not reached statistical significance merely because of the limited sample numbers.

Our study has several strengths. Firstly, we used clustered random sampling proportional to the Korean population and the estimated sampling error was low. Secondly, we investigated both anxiety and depression, which are common comorbid conditions among both the general population and individuals with migraine, and assessed clinical characteristics of migraine according to the status of anxiety and depression. Balancing limitations and strengths, we think that this study successfully assessed the association of anxiety and depression in migraine with their clinical implications.

## Conclusions

Approximately 1/3 and 1/6 of migraineurs had anxiety and depression in a Korean population-based sample, respectively. One-third of migraineurs with anxiety had depression and 2/3 of migraineurs with depression had anxiety. The concurrence of anxiety and depression was associated with higher headache frequency in migraineurs. Occurrence of anxiety was associated with exacerbation of headache intensity. Impact of headache increased with the accompaniment of anxiety and/or depression. The presence of anxiety and depression should be carefully evaluated in patients with migraine, in order to reduce the impact of headache as well as provide better treatment for these patients.

## Additional files

Additional file 1:
**STROBE check list (http://www.biomedcentral.com/content/supplementary/s12883-014-0238-4-s1.doc).**
Additional file 2:
**IRB/EC protocol approval letter (http://www.biomedcentral.com/content/supplementary/s12883-014-0238-4-s2.pdf).**
Additional file 3:
**Questionnaire-Korean (http://www.biomedcentral.com/content/supplementary/s12883-014-0238-4-s3.pdf).**
Additional file 4:
**Questionnaire-English (http://www.biomedcentral.com/content/supplementary/s12883-014-0238-4-s4.pdf).**

## Abbreviations

CM: Chronic migraine
DSM-IV TR: Manual of mental disorders-4 text revision
GAS: Goldberg anxiety scale
PHQ-9: Patient health questionnaire-9
VAS: Visual analogue scale
