# Erratum to: Combination of anxiety and depression is associated with an increased headache frequency in migraineurs: a population-based study

**DOI:** 10.1186/s12883-016-0571-x

**Published:** 2016-04-19

**Authors:** Kyungmi Oh, Soo-Jin Cho, Yun Kyung Chung, Jae-Moon Kim, Min Kyung Chu

**Affiliations:** Department of Neurology, Korea University Guro Hospital, Korea University School of Medicine, Seoul, Korea; Department of Neurology, Dongtan Sacred Heart Hospital, Hallym University College of Medicine, Hwaseong, Korea; Department of Occupational and Environmental Medicine, Sacred Heart Hospital, Hallym University College of Medicine, Anyang, Korea; Department of Neurology, Chungnam National University, College of Medicine, Daejeon, Korea; Department of Neurology, Sacred Heart Hospital, Hallym University College of Medicine, Anyang, Korea

## Erratum

After publication of the original article [1], the authors noticed that the article contained some errors in the data presented within Table 1, Figs. 1 and 2, and Tables 2 and 3.

**Fig. 1:**
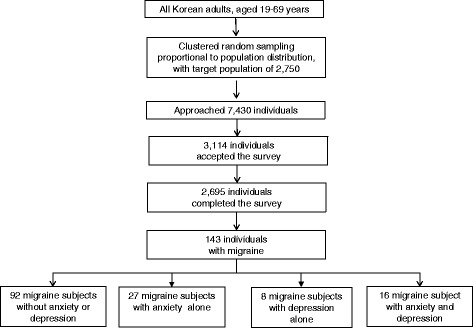
Flow chart depicting the participation of subjects in the Korean Headache-Sleep Study

**Fig. 2:**
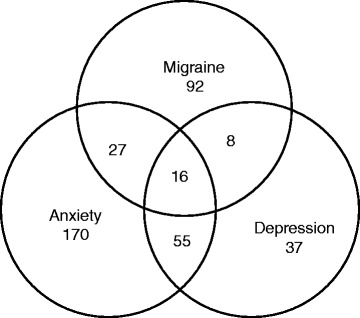
Venn diagram for distribution of subjects with anxiety, depression and migraine

**Table 1:** Sociodemographic distribution of all survey participants, the total Korean population, and of cases identified as migraine, anxiety and depression

	Survey participants *N* (%)	Total population *N* (%)	*P*	Migraine *N*, % (95 % CI)	Anxiety *N*, % (95 % CI)	Depression *N*, % (95 % CI)
Men	1345 (49.3)	17,584,365 (50.6)	0.854^a^	36, 2.7 (1.8–3.5)	109, 8.1 (6.6–9.6)	43, 3.2 (2.3–4.2)
Women	1350 (50.7)	17,198,350 (49.4)		107, 7.9 (6.5–9.4)	159, 11.8 (10.1–13.5)	73, 5.4 (4.2–6.6)
Age						
19–29	542 (20.5)	7,717,947 (22.2)	0.917^a^	25, 4.5 (2.7–6.2)	53, 9.6 (7.2–12.1)	23, 4.1 (2.5–5.8)
30–39	604 (21.9)	8,349,487 (24.0)		42, 7.0 (4.9–9.1)	51, 8.7 (6.4–11.0)	32, 5.4 (4.6–7.3)
40–49	611 (23.1)	8,613,110 (24.8)		39, 6.5 (4.5–8.4)	67, 11.0 (8.5–13.5)	24, 4.0 (2.5–5.5)
50–59	529 (18.9)	6,167,505 (17.7)		22, 4.1 (2.4–5.9)	53, 9.9 (7.3–12.5)	22, 4.2 (2.5–6.0)
60–69	409 (15.6)	3,934,666 (11.3)		15, 3.9 (2.0–5.7)	14, 10.8 (7.8–13.8)	15, 3.7 (2.0–5.5)
Size of residential area						
Large city	1248 (46.3)	16,776,771 (48.2)	0.921^a^	76, 6.1 (4.8–7.5)	130, 10.4 (8.7–12.1)	57, 4.6 (3.4–5.7)
Medium–to–small city	1186 (44.0)	15,164,345 (43.6)		48, 4.0 (2.9–5.2)	112, 9.5 (7.8–11.2)	47, 4.0 (2.9–5.1)
Rural area	261 (9.7)	2,841,599 (8.2)		19, 7.4 (4.2–10.6)	26, 10.0 (6.3–13.6)	12, 4.7 (2.1–7.3)
Education level						
Middle school or less	393 (14.9)	6,608,716 (19.0)	0.752^a^	22, 5.5 (4.2–7.7)	55, 13.9 (10.5–17.4)	20, 5.2 (3.0–7.4)
High school	1208 (44.5)	15,234,829 (43.8)		60, 5.0 (3.8–6.3)	111, 9.2 (7.5–10.8)	49, 4.1 (3.0–5.2)
College or more	1068 (39.6)	12,939,170 (37.2)		60, 5.6 (4.3–7.0)	100, 9.5 (7.7–11.2)	47, 4.4 (3.2–5.7)
Not responded	26 (9.6)			1, 3.8 (0.0–11.8)	2, 8.0 (0.0–18.0)	0, 0.0 (0.0–0.0)
Total	2695 (100.0)	34,782,715 (100.0)		143, 5.3 (4.5–6.2)	268, 10.0 (8.8–11.1)	116, 4.3 (3.6–5.1)

^a^Compared gender, age group, size of residential area, and educational level distributions between the sample of the present study and total population of Korea

**Table 2:** Demographics, headache characteristics and associated symptoms of migraineurs according to anxiety and depression

	Migraine subjects without anxiety or depression, *N* = 92	Migraine subjects with anxiety alone, *N* = 27	Migraine subjects with depression alone, *N* = 8	Migraine with anxiety and depression, *N* = 16	*P*-value
Demographics					
Mean age ± SD (years)	40.4 ± 11.4	47.2 ± 14.5	42.1 ± 15.7	44.5 ± 18.4	0.131
Women, *N* (%)	71 (75.5)	21 (77.8)	7 (77.8)	13 (76.5)	0.995
Headache characteristics					
Unilateral pain, *N* (%)	57 (60.6)	15 (53.6)	4 (44.4)	7 (41.2)	0.409
Pulsating quality, *N* (%)	72 (76.6)	21 (77.8)	7 (77.8)	12 (70.6)	0.950
Moderate-to-severe severity, *N* (%)	72 (75.8)	22 (81.5)	8 (88.9)	17 (100.0)	0.119
Aggravation by movement, *N* (%)	60 (63.8)	21 (77.8)	5 (55.6)	15 (88.2)	0.121
Associated symptoms					
Nausea, *N* (%)	84 (88.4)	23 (82.1)	9 (100.0)	15 (88.2)	0.544
Vomiting, *N* (%)	33 (34.7)	15 (53.6)	3 (33.3)	8 (47.1)	0.288
Photophobia, *N* (%)	55 (57.9)	19 (70.4)	1 (11.1)	11 (64.7)	0.017
Phonophobia, *N* (%)	63 (67.0)	21 (77.8)	6 (66.7)	13 (76.5)	0.668
Osmophobia, *N* (%)	42 (44.7)	13 (48.1)	4 (44.4)	9 (46.3)	0.930

**Table 3:** Frequency, severity and impact of headache according to migraineurs’ anxiety and depression status

	Migraine subjects without anxiety or depression, *N* = 92	Migraine subjects with anxiety alone, *N* = 27	Migraine subjects with depression alone, *N* = 8	Migraine subjects with anxiety and depression, *N* = 16	*P*-value*	Post hoc analysis with Bonferroni’s correction
	Median (25 %–75 %)	Median (25 %–75 %)	Median (25 %–75 %)	Median (25 %–75 %)		
Frequency per month	1.0 (0.3–3.0)	2.0 (1.0–5.0)	1.0 (0.3–4.0)	8.0 (2.5–21.0)	<0.001	1 vs. 2 = 0.596
						1 vs.3 = 1.000
						1 vs. 4 < 0.001
						2 vs.3 = 1.000
						2 vs. 4 = 0.003
						3 vs. 4 = 0.001
VAS score for pain intensity	6.0 (5.0–7.0)	7.0 (6.0–8.0)	7.0 (6.0–8.0)	7.0 (5.0–9.0)	<0.001	1 vs. 2 = 0.011
						1 vs.3 = 0.824
						1 vs. 4 = 0.018
						2 vs.3 = 1.000
						2 vs. 4 = 1.000
						3 vs. 4 = 1.000
HIT–6 score	50.0 (46.0–58.0)	57.0 (49.0–60.8)	62.0 (52.0–70.5)	64.0 (61.0–67.0)	<0.001	1 vs. 2 = 0.074
						1 vs.3 = 0.004
						1 vs. 4 < 0.001
						2 vs.3 = 0.545
						2 vs. 4 = 0.005
						3 vs. 4 = 1.000

HIT–6: Headache Impact Test–6; VAS: Visual Analogue Scale

*Kruskal–Wallis one–way analysis of variance test among the four groups: migraine subjects without anxiety or depression, migraine subjects with anxiety alone, migraine subjects with depression alone and migraine subjects with anxiety and depression

1: migraine subjects without anxiety or depression; 2: migraine subjects with anxiety alone; 3: migraine subjects with depression alone; 4: migraine with anxiety and depression

The corrected versions are given in this erratum. The main findings of the original article are not affected after this correction. The authors apologise for any inconvenience this has caused.
