# Supplementary material for: Combination of anxiety and depression is associated with an increased headache frequency in migraineurs: a population-based study
Source: BMC Neurol. 2014 Dec 14;14:238. doi: 10.1186/s12883-014-0238-4 (PMC4279894; doi:10.1186/s12883-014-0238-4)
Supplement: Additional file 2: — IRB/EC protocol approval letter (http://www.biomedcentral.com/content/supplementary/s12883-014-0238-4-s2.pdf). [file 12883_2014_238_MOESM2_ESM.pdf]

한림대학교성심병원
Hallym University Sacred Heart Hospital
896 Pyungchon, Anyang City 431-070, Korea
Tel) +82-31-380-1975 Fax) +82-31-381-4086

Protocol Approval Letter

IRB/EC of Hallym University Sacred Heart Hospital

DATE	25.Nov.2011
TO:	Min- Kyung, Chu
FROM	IRB/EC of Hallym University Sacred Heart Hospital
Protocol No:	
TITLE:	Epidemiology of sleepiness, sleep disorders and headache disorder in Korean population(The Korean Headache-Sleep Study)
NUMBER:	2011-I077

The protocol was approved by IRB/EC of Hallym University Sacred Heart Hospital on 25.Nov.2011. IRB/EC of Hallym University Sacred Heart Hospital is responsible for assigning the protocol number and maintaining the official record for all intramural on-site and off-site protocols. Your protocol number is 2011-I077, and will be due for continuing review on 25.Nov.2011~20.Aug.2013.

The investigator should report promptly any unanticipated problems involving risks to subjects or others, or serious harm involving subjects, to IRB/EC of Hallym University Sacred Heart Hospital. In addition, substantive changes in research activities, during the period for which IRB/EC approval has been given, may not be initiated without prior review and approval by IRB/EC, except where necessary to eliminate apparent immediate hazard to subjects.

If there are any questions regarding protocol review, approval, or reporting procedures, please contact Sun-Hyang Kim at [+82-31-380-1975].

Yoo, Sang-Ho, M.D.
Chairperson
Institutional Review Board/ Ethics Committee
Hallym University Sacred Heart Hospital
