# Supplementary material for: Combination of anxiety and depression is associated with an increased headache frequency in migraineurs: a population-based study
Source: BMC Neurol. 2014 Dec 14;14:238. doi: 10.1186/s12883-014-0238-4 (PMC4279894; doi:10.1186/s12883-014-0238-4)
Supplement: Additional file 3: — Questionnaire-Korean (http://www.biomedcentral.com/content/supplementary/s12883-014-0238-4-s3.pdf). [file 12883_2014_238_MOESM3_ESM.pdf]

110-054 종로구 사직동 208 전화 02-3702-2100, 팩스 02-3702-2121, 한국갤럽홈페이지 www.gallup.co.kr, 갤럽패널홈 panel.gallup.co.kr
한국갤럽 GALLUP KOREA *affiliated with GALLUP INTERNATIONAL*

A1-5

Gallup Omnibus 2011-310-014

2011 12월 옴니버스 조사

?
저는 한국갤럽조사연구소에서 조사원으로 일하는 ○○○입니다.
이번 저희 연구소에서는 **과 관련된 여러 가지 문제에 대한 의견**을 알아보고 있습니다.
제가 묻게 되는 질문에는 맞고 틀리는 답이 없으며, ○○ 님의 의견은 이런 의견을 갖고 있는 사람이 몇 퍼센트 (%)라는 식으로 통계를 내는 데에만 사용되며, 그 외의 목적에는 절대로 사용되는 일이 없으니 느끼시는 대로 말씀해 주시면 됩니다.
잠시만 시간을 내어 협조해 주시면 감사하겠습니다.

■ 지 역 : 6-7 □□

01) 울	02) 부 산	03) 대 구	04) 인 천	05) 광 주	06) 대 전
07) 울 산	08) 경 기	09) 강 원	10) 충 북	11) 충 남	12) 전 북
13) 전 남	14) 경 북	15) 경 남			

■ 지점번호 : 8-10 □□□

■ 지역크기 : 11 1. 2. 중소 도시 3. 읍 4. 면

■ 성 별 : 12 1. 남성 2. 여성

2011 12월
한 국 갤 럽 조 사 연 구 소
소 장 박 무 익
담당연구원 정 지 연 / 장 덕 현
실사연구원 박 주 희
주 소 서울시 종로구 사직동 208
전 화 02-3702-2681

SQ 3) 실례지만 ○○ 님의 **나이(연세)는 올해 만으로** 어떻게 되십니까? 생일을 고려하지 않고, 우리나라 나이에서 한 살을 뺀 만 나이로 응답해 주십시오.

올해 만 나이와 출생년도를 모두 적어 주십시오.

13-14 만 □□세 (19□□년생) 15-16

SQ 4) 조사원 다음은 질문하지 말고 SQ3)에 따라 체크하십시오.
연령대

17
1. 만 19 - 29세
2. 만 30 - 39세
3. 40 - 49세
4. 만 50 - 59세
5. 만 60세 이상

SQ 5) 조사원 다음은 질문하지 말고 체크하십시오.
조사 지역

18
1. 인접 동 등 지정 동 이외 지역 진행
2. 지정 동 내 진행

SQ 6) ○○ 님의 **직업**은 무엇입니까?
19-20

1. 농업 · 수산업 · 축산업(가족종사자 포함)
2. (종업원 9명 이하의 소규모 업소 주인 및 가족 종사자, 약국, 개인택시운전사)
3. 판매직 · 서비스직(상점점원, 세일즈맨, 보험설계사 등)
4. 기능공 · 숙련공(중장비 · 트럭 운전사, 전자 · 가전제품 A/S 기술자, 숙련공 등)
5. 일반작업직(토목 관계의 현장 작업, 청소, 수위, 육체 노동 등)
6. 사무직 · 기술직(일반회사 사무직, 기술직, 유치원 · 학교 교사, 회사에 소속된 웹디자이너 · 컴퓨터 프로그래머 등)
7. 경영관리직(5급 이상의 고급공무원, 교장, 기업체 부장 이상의 직위 등)
8. 전문직(대학교수, 변호사, 의사, 회계사, 종교인, 언론인, 예술가, 고소득 프리랜서 등)
9. 가정주부
10. 학생(고등학생, 대학생, 대학원생)
11. 무직
12. 은퇴
13. 기타 (적을 것: ____________________)

Copyright(c) 2011 Gallup Korea Ltd. All rights reserved.

'두통'에 대한 질문입니다.

19) ○○ 님께서는 __1년간 두통을 1분 이상 경험__하신 적이 있으십니까?

B65

1.
2. 아니오 ──▶ 6 문 29)로 가십시오

문 20) ○○ 님께서 경험하신 두통은 평균적으로 얼마나 오래 갔습니까? 두통 때마다 약을 드신다면 약을 복용하지 않은 상태에서 두통이 지속된 시간을 말씀해 주십시오.

평균 ☐☐시간 ☐☐분

66-67 68-69

문 21) ○○ 님께서는 평소 두통을 얼마나 자주 경험하십니까? 다음 중 ○○ 님께서 두통을 경험하시는 빈도를 가장 잘 표현하는 항목을 한 가지만 골라 구체적으로 해당 기간 동안 몇 회 정도 경험하시는지를 말씀해 주십시오.

1. 1년에 ( )회 70-71
2. 1달에 ( )회 72-73
3. 1주일에 ( )회 74-75
4. 1일에 ( )회 76-77
5. 1시간에 ( )회 78-79

문 22) **(보기카드 6 제시)**
○○ 님께서 경험하신(혹은 느끼고 계신) 두통은 어느 정도였습니까? 다음 중에서 말씀해 주십시오.

80
1. 두통이 있기는 하지만 일상 활동에 지장을 주지는 않는 정도
2. 두통으로 일상 활동에 지장이 있기는 하지만 할 수 있는 정도
3. 두통으로 인하여 일상 활동을 할 수 없는 정도

문 23) **(보기카드 7 제시)**
그럼, 두통 정도를 '통증이 없는 상태' 0점, '상상할 수 있는 가장 심한 통증' 10점으로 볼 때 ○○ 님께서 경험하신 두통은 평균적으로 1점에서 10점 사이에서 어느 정도인지 말씀해 주십시오.

C6-7

없음										가장 심한 통증
0	1	2	3	4	5	6	7	8	9	10

문 24) ○○ 님께서는 두통을 느끼실 때 머리 어느 부위가 특히 아프십니까? **(단수응답)**

8-9
1. 오른쪽
2. 왼쪽
3. 양쪽
4. 머리 전체
5. 번갈아가며 한 쪽만
6. 아플 때 마다 여기저기 부위가 바뀐다
7. 기타(적을 것: ______________________)

문 25) ○○ 님께서 느끼신 두통의 증세는 어떠했습니까? ○○ 님께서 두통을 경험하실 때의 느낌을 구체적인 말로 표현해 주십시오.**(자유응답)**

10-12 ______________________________

문 26) **(보기카드 8 제시)**
그럼, ○○ 님께서 __지난 1년간__ 경험하신 두통 증세에 맞는 내용을 다음 중에서 __모두__ 말씀해 주십시오.**(복수응답)**

13 1. 심장 뛰듯이 욱신거리거나 지끈거린다
14 2. 머리가 터질 것 같이 아프다
15 3. 머리가 누르거나 조이거나 또는 띠를 두른 것 같다
16 4. 바늘로 순간적으로 콕콕 찌르듯이 아프다
17 5. 망치로 머리를 내려치는 것 같이 순간적으로 심한 두통이 발생한다
18-19 6. 기타(적을 것: ______________________)

문 27) **(보기카드 9 제시)**
○○ 님께서는 다음 각 증세를 경험한 적이 있으십니까, 혹은 없으십니까?

[redacted] ① ~ ⑬ 각각에 대해 질문하시고, ⑫와 ⑬ 증세 경험자에게는 지난 3개월간 총 경험일수도 질문하십시오.

증세	예	아니오	경험 일수
① 체하면 머리가 아프다 20	1	2	
② 두통이 있을 때 속이 메슥거린다 21	1	2	
③ 두통이 있을 때 구토가 나타난다 22	1	2	
④ 평소 아무렇지 않게 느껴졌던 빛이나 밝은 곳이 거슬리거나 불편하게 느껴진다 23	1	2	
⑤ 머리가 아플 때 움직이거나 계단을 걷는 것과 같은 일상적인 활동에 의해 두통이 악화된다 24	1	2	
⑥ 두통이 있을 때 평소 아무렇지 않게 느껴졌던 소음들이 불편하게 들린다 25	1	2	
⑦ 두통이 있을 때 평소 아무렇지 않게 느껴졌던 냄새가 다르거나 불편하게 느껴진다 26	1	2	
⑧ 두통이 있거나 있기 몇 분 전에 눈앞에 불빛이 번쩍거리거나, 별 같은 것이 보이거나, 지그재그로 형태의 선이 보이거나, 사물의 일부가 흐릿하게 보이지 않게 되는 현상이 있다 27	1	2	
⑨ 두통이 있을 때나 있기 전에 어지럼을 느낀다 28	1	2	
⑩ 망치로 두들겨 맞은 듯한 심한 두통이 갑자기 시작된 적이 있었다 29	1	2	
⑪ 머리 한쪽으로 나타나는 심한 두통이 하루 4시간 이하로 거의 매일 연속해서 7일 이상 나타난 적이 있다 30	1	2	
⑫ __지난 3개월 동안__ 두통 때문에 직장이나 학교에서 결근, 결석하거나 집안일을 하지 못한 적이 있다 31	1	2	3개월 중 ____일 33-34
⑬ __지난 3개월 동안__ 두통 때문에 학교나 직장에서 업무에 장애가 있거나, 집안일을 잘 하지 못한 적이 있다 32	1	2	지난 3개월 중 ____일 35-36

Copyright(c) 2011 Gallup Korea Ltd. All rights reserved.

28) (10 제시)
○○ 님께서는 다음 각 항목을 얼마나 자주 경험하십니까? 각 항목별로 '한 번도 그런 적이 없다', '드물게 그렇다', '때때로 그렇다', '자주 그렇다', '항상 그렇다' 중에서 말씀해 주십시오.

번도 그런 적이 없다	드물게 그렇다	때때로 그렇다	자주 그렇다	항상 그렇다
1	2	3	4	5

① 두통이 있을 때, 통증이 심하다고 느낀다	1	2	3	4	5	C37
② 두통 때문에 집안일, 직장일, 학교 또는 사회활동 등 일상생활에 지장을 받는다	1	2	3	4	5	38
③ 두통이 있을 때 누워서 쉬고 싶을 때가 있다	1	2	3	4	5	39
④ <u>지난 4주 동안</u>, 두통 때문에 일이나 일상생활을 못할 정도로 피곤했다	1	2	3	4	5	40
⑤ <u>지난 4주 동안</u>, 두통 때문에 짜증이나 신경질이 났다	1	2	3	4	5	41
⑥ <u>지난 4주 동안</u>, 두통 때문에 일이나 일상생활에 집중하기 힘들었다	1	2	3	4	5	42

[redacted] 응답자에게 질문

문 29) **(보기카드 11 제시)**
○○ 님 가족이나 친척들 중에 두통으로 고생하시는 분이 계십니까? **(계시면)** 다음 중 해당되는 분을 <u>모두</u> 말씀해 주십시오.**(복수응답)**

43 1.
44 2. 할머니
45 3. 아버지
46 4. 어머니
47 5. 남자 형제
48 6. 여자 자매
49 7. 자식
50 8. 사촌 형제 자매
51 9. 삼촌
52 10. 고모
53 11. 외삼촌
54 12. 이모
55 13. 조카
56-57 14. 기타(적을 것: ____________________)

58 99. 두통으로 고생하는 가족/친척이 없다

문 30) 다음은 사람들이 자신을 표현하는데 사용하는 내용입니다. 각 항목에 대해 ○○ 님의 경우를 '그렇다', '아니다'로 응답해 주십시오.

내용	그렇다	아니다	
① 나는 절벽에 서 있는 기분이다	1	0	59
② 나는 걱정을 많이 하는 편이다	1	0	60
③ 나는 쉽게 짜증을 낸다	1	0	61
④ 나는 마음을 가라앉히기가 어렵다	1	0	62
⑤ 나는 잠을 제대로 자지를 못한다	1	0	63
⑥ 나는 머리가 아프거나 목이 뻐근하다	1	0	64
⑦ 나는 몸이 떨리거나, 저리거나, 갑자기 어지럽거나, 땀이 나거나, 소변이 자주 마렵고, 설사를 하는 등의 증세 중 한 가지 이상이 있다	1	0	65
⑧ 나는 건강에 대하여 걱정을 많이 한다	1	0	66
⑨ 나는 잠들기가 어렵다	1	0	67

귀하의 지난 한 달 평상시 수면 습관에 대해 여쭙겠습니다.

문 31) <u>지난 한 달 동안</u>, 평균 몇 시에 잠자리에 드셨습니까? 주중과 휴일 기준으로 각각 말씀해주십시오.

① 주중 (오전/오후 ________시 ________분)
68 69-70 71-72
② 휴일 (오전/오후 ________시 ________분)
73 74-75 76-77

문 32) <u>지난 한 달 동안</u>, 평균 몇 시에 잠자리에서 일어나셨습니까? 주중과 휴일 기준으로 각각 말씀해주십시오.

① 주중 (오전/오후 ________시 ________분)
78 79-80 81-82
② 휴일 (오전/오후 ________시 ________분)
83 84-85 86-87

문 33) <u>지난 한 달 동안</u>, 하루 평균 몇 시간 정도 주무셨습니까? 주중과 휴일 기준으로 각각 말씀해주십시오.

① 주중 (________시간 ________분)
D6-7 8-9
② 휴일 (________시간 ________분)
10-11 12-13

문 34) ○○ 님께서는 본인의 수면 시간이 충분하다고 생각하십니까, 혹은 충분하지 않다고 생각하십니까?

14
1. 충분하다 ▶ 7페이지 문 36)으로 가십시오
2. 충분하지 않다

문 35) **(충분하지 않다면)**
○○ 님께서 원하시는 수면 시간은 몇 시간 정도입니까?

________시간 ________분 정도
15-16 17-18

Copyright(c) 2011 Gallup Korea Ltd. All rights reserved.

[redacted] 응답자에게 질문

36) 한 달 동안, 잠이 오지 않아 수면제를 복용하신 경험이 있습니까, 혹은 없습니까?(처방약 또는 약국에서 구입한 약)

D19
1.
2. 없다 ▶ 문 38)로 가십시오

문 37) 그럼, 지난 한 달 동안 잠들기 위해 얼마나 자주 약을 드셨습니까?(처방약 또는 약국에서 구입한 약)

20
1. 한 주에 한 번보다 적게
2. 한 주에 한두 번 정도
3. 한 주에 세 번 이상

조사원 | 모든 응답자에게 질문

문 38) **(보기카드 12 제시)**
일상생활 중 지나치게 졸리면 어떻게 대응하십니까?
다음 중에서 모두 말씀해 주십시오.**(복수응답)**

21 1. 커피 및 카페인 음료를 마신다
22 2. 담배를 피운다
23 3. 운동을 한다
24 4. 낮잠(토막잠)을 잔다
25-26 5. 기타(적을 것: ______________________)
27 6. 없다

조사원 | 문 39)와 문 40)은 SQ3의 가정주부, 무직, 은퇴를 제외하고 질문. 그 외의 분들은 문 41)로 가십시오.

문 39) ○○ 님께서는 평소 몇 시에 직장(일터)이나 학교에 출근/등교하십니까?

(오전 / 오후) ________시 ________분
28 29-30 31-32

문 40) ○○ 님은 교대 근무를 하십니까?

33
1. 예
2. 아니오

조사원 | 모든 응답자에게 질문

문 41) 지난 3개월 동안, 안전 부주의로 인한 사고 경험이 있습니까, 혹은 없습니까? 가벼운 안전 사고도 포함하여 말씀해 주십시오.

34
1. 있다
2. 없다

문 42) **(보기카드 13 제시)**
다음 상황에서 ○○ 님께서 단지 피곤함을 느끼는 것이 아니라 졸리거나 잠이 들 가능성이 얼마나 됩니까?
평상시의 생활을 생각하시고 말씀해 주십시오.
최근 다음과 같은 상황이 없었더라도, 그런 상황에 놓이면 어떻게 될 지 가정해서 응답해 주십시오.

아래의 점수를 기준으로 각 상황에서 가장 적당한 점수를 말씀해 주십시오.

졸지 않는다	약간 졸 가능성이 있다	중간 정도로 졸 가능성이 있다	거의 매번 졸게 된다
0	1	2	3

① 앉아서 책을 읽을 때	0	1	2	3	35
② TV 시청할 때	0	1	2	3	36
③ 공공 장소에서 가만히 앉아있을 때 (극장, 모임 등)	0	1	2	3	37
④ 승객으로 한 시간 동안 멈추지 않고 차를 타고 갈 때	0	1	2	3	38
⑤ 오후 시간에 짬이 나 누워서 휴식을 취할 때	0	1	2	3	39
⑥ 앉아서 누군가와 대화를 나눌 때	0	1	2	3	40
⑦ 술이 없는 점심 식사 후 조용히 앉아있을 때	0	1	2	3	41
⑧ 운전 중에 교통이 정체되어 몇 분간 멈춰 서 있을 때	0	1	2	3	42

문 43) **(보기카드 14 제시)**
지난 한 달 동안 다음 이유로 잠자는데 얼마나 자주 문제가 있었습니까?
'한 번도 없었다', '한 주에 한 번보다 적게', '한 주에 한두 번 정도', '한 주에 세 번 이상' 중에서 말씀해 주십시오.

한 번도 없었다	한 주에 한 번보다 적게	한 주에 한두 번 정도	한 주에 세 번 이상
1	2	3	4

① 30분 이내로 잠들 수 없다	1	2	3	4	43
② 한밤중이나 새벽에 깬다	1	2	3	4	44
③ 화장실에 가려고 일어난다	1	2	3	4	45
④ 편안하게 숨 쉴 수 없다	1	2	3	4	46
⑤ 기침을 하거나 시끄럽게 코를 곤다	1	2	3	4	47
⑥ 심한 추위를 느낀다	1	2	3	4	48
⑦ 심한 더위를 느낀다	1	2	3	4	49
⑧ 나쁜 꿈을 꾼다	1	2	3	4	50
⑨ 통증이 있다	1	2	3	4	51
⑩ 그 외에 잠자는데 다른 문제가 있다면 말씀해 주십시오. ______________ 52-53					
이 이유 때문에 얼마나 자주 잠자는데 어려움이 있었습니까?	1	2	3	4	54

Copyright(c) 2011 Gallup Korea Ltd. All rights reserved.

44) 한 달 동안 잠자리에 드셔서 실제로 잠이 들 때까지 걸린 시간은 평균 어느 정도입니까?

______ ______ 분
D55-56 57-58

문 45) **지난 한 달 동안** 운전하거나, 식사하실 때, 또는 사회 활동을 하는 동안 **얼마나 자주** 졸음을 느끼셨습니까?

59 1. 번도 없었다
2. 한 주에 한 번보다 적게
3. 한 주에 한두 번 정도
4. 한 주에 세 번 이상

문 46) **지난 한 달 동안**, 하는 일에 열중하는데 얼마나 많은 어려움(문제)이 있었습니까?

60 1. 전혀 문제 없었다
2. 약간의 문제가 있었다
3. 상당히 문제가 있었다
4. 매우 큰 문제가 있었다

문 47) **지난 한 달 동안**, ○○ 님의 전반적인 수면의 질은 어느 정도라고 평가하십니까?

61 1. 매우 좋다
2. 약간 좋다
3. 약간 나쁘다
4. 매우 나쁘다

문 48) **(보기카드 15 제시)**
지난 2 주 동안, 아래 나열되는 증상들을 얼마나 자주 경험하셨습니까? '전혀 없음', '여러 날 동안', '일주일 이상', '거의 매일' 중에서 말씀해 주십시오.

없음	여러 날 동안	일주일 이상	거의 매일
0	1	2	3

① 일을 하는 것에 대한 흥미나 재미가 거의 없음	0	1	2	3	62
② 가라앉은 느낌, 우울감 혹은 절망감	0	1	2	3	63
③ 잠들기 어렵거나 자꾸 깨어남, 혹은 너무 많이 잠	0	1	2	3	64
④ 피곤, 기력이 저하됨	0	1	2	3	65
⑤ 식욕 저하 혹은 과식	0	1	2	3	66
⑥ 내 자신이 나쁜 사람이라는 느낌 혹은 내 자신을 실패자라고 느끼거나, 나 때문에 나 자신이나 내 가족이 불행하게 되었다는 느낌	0	1	2	3	67
⑦ 신문을 읽거나 TV를 볼 때 집중하기 어려움	0	1	2	3	68
⑧ 남들이 알아챌 정도로 거동이나 말이 느림 또는 반대로 너무 초조하고 안절부절 못해서 평소보다 많이 돌아다니고 서성거림	0	1	2	3	69
⑨ 나는 차라리 죽는 것이 낫겠다는 등의 생각 혹은 어떤 면에서건 나 스스로에게 상처를 주는 생각들	0	1	2	3	70

[redacted] 49)는 문 48)에서 한 개라도 0점이 아닌 증상이 있는 응답자에게만 질문하고, 모두 0점이면 문 50)으로 가십시오.

문 49) 응답하신 증상(들) 때문에 직장이나 집안일을 하거나 주변 사람들과의 관계에서 얼마나 어려움을 겪으셨습니까?

71 1. 전혀 어려움이 없었다
2. 별로 어려움이 없었다
3. 어느 정도 어려움이 있었다
4. 매우 어려움이 있었다

조사원 | 모든 응답자에게 질문

문 50) ○○ 님에게는 **불면증**이 있습니까?

72 1. 있다
2. 없다 → 문 52)로 가십시오

문 51) **(보기카드 16 제시)**
그럼, 얼마나 자주 불면증을 경험하시나요?
1주일 기준으로 말씀해 주십시오.

73 1. 1주일에 1일 미만
2. 1주일에 1~2일 정도
3. 1주일에 3~4일 정도
4. 1주일에 5일 이상

조사원 | 모든 응답자에게 질문

문 52) **(보기카드 17 제시)**
지난 2주 동안, '잠'에 관한 다음 문제들이 얼마나 심하셨는지, '그렇지 않음', '약간', '중간', '어느 정도 심함', '매우 심함' 중에서 말씀해 주십시오.

잠 관련 문제	그렇지 않음	약간	중간	어느 정도 심함	매우 심함	
① 잠들기 어렵다	0	1	2	3	4	74
② 잠을 유지하기 어렵다	0	1	2	3	4	75
③ 일찍 잠이 깬다	0	1	2	3	4	76
④ 자고 일어나도 개운하지 않다	0	1	2	3	4	77

문 53) ○○ 님께서는 자신의 수면 양상에 대해 얼마나 만족하십니까, 혹은 불만족하십니까?

78 1. 매우 만족
2. 약간 만족
3. 보통이다
4. 약간 불만족
5. 매우 불만족

Copyright(c) 2011 Gallup Korea Ltd. All rights reserved. |

54) (18 제시)
○○ 님의 수면 문제가 어느 정도나 ○○ 님의 낮 활동을 방해한다고 생각합니까? (예: 낮에 피곤함, 직장/학교에서
D79 일하는 능력, 집중력, 기억력, 기분 등)

방해되지 않는다	약간	다소	상당히	매우 많이 방해한다
1	2	3	4	5

문 55) (보기카드 19 제시)
다른 사람에게 ○○ 님의 수면 문제로 인해서 ○○ 님의 삶의 질이 어느 정도나 손상되는 것으로 보일 것이라고
80 생각하십니까?

전혀 손상되어 보이지 않을 것	약간	다소	상당히	매우 심하게 손상된 것으로 보일 것
1	2	3	4	5

문 56) (보기카드 20 제시)
○○ 님은 현재 ○○ 님의 수면 문제에 관하여 얼마나
81 걱정하십니까?

전혀 걱정하지 않는다	약간	다소	상당히	매우 많이 걱정한다
1	2	3	4	5

문 57) (보기카드 21 제시)
○○님 가족이나 친척들 중에 불면증으로 고생하시는 분이 계십니까? **(계시면)** 다음 중에서 해당되는 분을 <u>모두</u> 말씀해 주십시오.**(복수응답)**

E6 1.
7 2. 할머니
8 3. 아버지
9 4. 어머니
10 5. 남자 형제
11 6. 여자 자매
12 7. 자식
13 8. 사촌 형제 자매
14 9. 삼촌
15 10. 고모
16 11. 외삼촌
17 12. 이모
18 13. 조카
19-20 14. 기타(적을 것: ________________)

21 99. 불면증으로 고생하는 가족/친척이 없다

문 58) ○○ 님께서는 주무실 때 코를 곱니까?
22
1. 예
2. 아니오 ──▶ 63)으로 가십시오
3. 모르겠다

문 59) 코고는 소리가 얼마나 심합니까?

23
1. 숨쉬는 소리보다 약간 더 크다
2. 대화하는 소리만큼 크다
3. 대화하는 소리보다 더 크다
4. 아주 시끄럽다(옆방에서 들리는 정도다)

문 60) 얼마나 자주 코를 곱니까?

24
1. 거의 매일
2. 1주일에 3~4회
3. 1주일에 1~2회
4. 한 달에 1~2회

문 61) ○○ 님의 코골이가 다른 사람의 수면을 방해한다고 생각하십니까?

25
1. 예
2. 아니오
3. 모르겠다

문 62) 주위 분들로부터 ○○ 님이 '수면 도중 숨을 안 쉬는 경우가 있다'는 말을 들으신 적이 있습니까?
(있다면) 그런 경우가 얼마나 자주 있습니까?

26
1. 거의 매일
2. 1주일에 3~4회
3. 1주일에 1~2회
4. 한 달에 1~2회
5. 거의 들어본 적이 없다

조사원 응답자에게 질문

문 63) 아침에 잠에서 깨면 피로를 느끼는 경우가 얼마나 자주 있습니까?

27
1. 거의 매일
2. 1주일에 3~4회
3. 1주일에 1~2회
4. 한 달에 1~2회
5. 거의 또는 전혀 피로하지 않다

문 64) 그럼, 낮에 깨어 있을 때는 피로를 느끼는 경우가 얼마나 자주 있습니까?

28
1. 거의 매일
2. 1주일에 3~4회
3. 1주일에 1~2회
4. 한 달에 1~2회
5. 거의 또는 전혀 피로하지 않다

Copyright(c) 2011 Gallup Korea Ltd. All rights reserved. |

65) 차를 운전하다 깜빡 존 적이 있습니까?

E29
1.
2. 아니오
3. 운전하지 않음 —— ▶ 67)으로 가십시오

문 66) 그런 적이 있다면, 얼마나 자주 그렇습니까?

30
1. 거의 매일
2. 1주일에 3~4회
3. 1주일에 1~2회
4. 한 달에 1~2회
5. 한 달에 1~2회보다 적게

조사원 | 응답자에게 질문

문 67) **질문에는 평소 느끼신 대로 응답해 주십시오.**
가만히 앉아 있거나 누워있을 때, 반복적으로 다리에 불편한 느낌(감각)을 경험하신 적이 있습니까?

31
1. 예
2. 아니오

문 68) 가만히 앉아 있거나 누워있을 때, 반복적으로 다리를 움직일 필요나 충동을 느끼신 적이 있습니까?

32
1. 예
2. 아니오

조사원 | 문 69)부터 문 79)까지는 문~~46)~~67), 문~~47)~~68) 중 하나라도 '1. 예', 즉 다리에 문제를 경험한 사람에게만 질문하고 둘 다 '2. 아니오'인 경우는 11페이지 문 80)으로 가십시오.

위에서 언급된 다리의 '느낌'에 관한 질문입니다

조사원 | 문 67), 문 68)에서 다리 문제 경험자에게만 질문

문 69) 가만히 있을 때(앉거나 누워있기)와 몸을 움직일 때 중, 언제 더 느낍니까?

33
1. 가만히 있을 때
2. 움직일 때

문 70) 일어나거나 움직이면, 움직임을 지속하는 동안에는 이런 느낌이 조금이라도 나아집니까?

34
1. 예
2. 아니오
3. 모르겠다

문 71) 하루 중 언제 이런 느낌이 가장 잘 생깁니까? '아침, 정오, 오후, 저녁, 밤' 중에서 <u>모두</u> 말씀해 주십시오.**(복수응답)**

35 1. 아침
36 2. 정오
37 3. 오후
38 4. 저녁
39 5. 밤
40 6. 하루 종일 비슷하다

문 72) 계속 움직이지 않더라도 다리 위치를 한번 바꾸면 이런 느낌이 좋아집니까?

41
1. 대개 그렇다
2. 대개 그렇지 않다
3. 모르겠다

문 73) 이런 느낌이 다리 근육경련(쥐) 때문에 생긴 적이 있습니까?

42
1. 예
2. 아니오 ▶ 문 75)로 가십시오
3. 모르겠다

문 74) 그렇다면, 이런 느낌이 항상 다리 근육경련(쥐) 때문에 생깁니까?

43
1. 예
2. 아니오
3. 모르겠다

문 75) 이런 느낌이 가만히 앉아 있을 때와 누워있을 때 중에서 언제 생깁니까?

44
1. 앉아있을 때만
2. 누워있을 때만
3. 앉거나 누워 있을 때 모두
4. 두 경우 모두 증상이 없다

문 76) 실제 다리에 이런 느낌이 들 때, 얼마나 불편합니까?

45
1. 별로 불편하지 않다
2. 약간 불편하다
3. 중간정도 불편하다
4. 매우 불편하다

문 77) <u>지난 1년 동안</u> 다리에 이런 느낌이 얼마나 자주 있었습니까?

46
1. 매일
2. 일주일에 4~5일
3. 일주일에 2~3일
4. 일주일에 1일
5. 한 달에 2일
6. 한 달에 1일 또는 그 미만
7. 한 번도 없었다

문 78) 처음 이런 느낌이 생긴 것은 몇 살 무렵이었습니까?

47-48 ____________세

문 79) **(보기카드 21 제시)**

Copyright(c) 2011 Gallup Korea Ltd. All rights reserved. |

○○ 가족이나 친척들 중에 ○○ 님이 느끼시는 다리 불편감으로 고생하시는 분이 계십니까?
( ) 다음 중 해당되는 분을 <u>모두</u> 말씀해 주십시오.
(복수응답)

E49 1.
50 2. 할머니
51 3. 아버지
52 4. 어머니
53 5. 남자 형제
54 6. 여자 자매
55 7. 자식
56 8. 사촌 형제 자매
57 9. 삼촌
58 10. 고모
59 11. 외삼촌
60 12. 이모
61 13. 조카
62-63 14. 기타(적을 것: ______________________)

64 99. 다리불편감으로 고생하는 가족/친척이 없다

응답자에게 질문

문 80) **지금부터는 평소 건강과 생활습관에 대한 질문입니다.**
○○ 님께서는 평소에 술을 드십니까? 혹은 현재 끊으신 상태입니까, 아니면 원래 술을 안 드십니까?
(술을 드신다면) 얼마나 자주 드십니까?

65
1. 거의 매일
2. 1주일에 4~6회 정도
3. 1주일에 2~3회 정도
4. 1주일에 1회 정도
5. 한달에 2~3회 정도
6. 한달에 1회 정도

7. 술을 끊었다
8. 원래 안 마신다

문 81) ○○ 님께서는 평소에 담배를 피우십니까? 혹은 현재 끊으신 상태입니까, 아니면 원래 담배를 안 피우십니까?

66
1. 평소에 담배를 피운다
2. 담배를 끊었다
3. 원래 피우지 않는다

문 82) ○○ 님께서는 평소에 몸에서 땀이 날 정도의 운동을
75 하십니까? **(하신다면)** 얼마나 자주 운동을 하십니까?

67
1. 거의 매일
2. 1주일에 5~6일 정도
3. 1주일에 3~4일 정도
4. 1주일에 1~2일 정도

5. 운동을 하지 않는다

문 83) **(보기카드 22 제시)**

다음 중 앓으셨거나 앓고 계신 질병을 <u>모두</u> 말씀해 주십시오. **(앓은 질병 각각에 대해)** 그 질병은 몇 세 때 처음 앓으셨습니까?

	발병 여부		최초 발병 나이	
① 뇌졸중 (중풍/뇌경색/뇌출혈)	1	68	____세	69-70
② 심근경색	2	71	____세	72-73
③ 말초혈관질환	3	74	____세	75-76
④ 협심증	4	77	____세	78-79
⑤ 고혈압	5	80	____세	81-82
⑥ 당뇨	6	83	____세	84-85
⑦ 고지혈증	7	86	____세	87-88
⑧ 심장병	8	89	____세	90-91
⑨ 모두 앓은 적 없음	9	92		

문 84) 앞에서 제시된 질병 외에 **현재 앓고 계신 다른 질병**이 있습니까? **(있다면)** 어떤 질병인지 <u>모두</u> 말씀해 주십시오.

F6-8 ① ______________________
9-11 ② ______________________
12-14 ③ ______________________

999. 다른 질병이 없다

문 85) 실례지만, ○○ 님의 키와 몸무게는 얼마입니까?
조사원 소수점 이하는 반올림해서 정수로 적어주십시오.

15-17 키 : ☐☐☐ cm
18-20 몸무게 : ☐☐☐ kg

분류용 질문

질문에 응답해 주셔서 고맙습니다.
마지막으로 자료 분류를 위해 몇 가지만 더 여쭙겠습니다.
이 항목들은 통계적인 자료 분류 목적 이외에는
결코 사용하지 않을 것을 약속 드립니다.

D 6) 실례지만, ○○ 님은 **학교**를 어디까지 마치셨습니까?
조사원 중퇴는 졸업에 포함되지 않습니다.

26
1. 초등학교 졸업 이하
2. 중학교 졸업
3. 고등학교 졸업
4. 대학 재학/졸업
5. 대학원 재학 이상

D 12-2) 그럼, ○○ 님의 **한달 평균 총수입**은 얼마 정도입니까?
임대소득, 예금이자 등을 모두 포함한 개인 월평균 총수입을 말씀해 주십시오.

1. 월 49만원 이하
9. 월 400 ~ 499만원

Copyright(c) 2011 Gallup Korea Ltd. All rights reserved. |

2.   50 ~ 99만원
3. 월 100 ~ 149만원
4. 월 150 ~ 199만원
5. 월 200 ~ 249만원
6. 월 250 ~ 299만원
7. 월 300 ~ 349만원
8. 월 350 ~ 399만원
10. 월 500 ~ 599만원
11. 월 600 ~ 699만원
12. 월 700 ~ 799만원
13. 월 800 ~ 899만원
14. 월 900 ~ 999만원
15. 월 1,000만원 이상
16. 소득 없음

향후 조사 참여 의향이 있는 분에게는 연락처를 좀 더
정확하게 확인하시고, 빠짐없이 기록해 주십시오.
단, 거절자인 경우에도 면접후기록은 반드시 작성하셔야 합니다.

접 후 기 록

이름	
응답자 주소	
응답자연락처	1. ( ) - ( ) - ( ) *43-45* *46-49* *50-53*<br>국 번호<br>2. 휴대폰 ( ) - ( ) - ( ) *54-56* *57-60* *61-64*
조 사 일 시	______월 ______일 *65-66* *67-68*<br>______시 ______분부터 ______시 ____분까지<br>(분간) *69-70*<br>(실제 응답 소요 시간을 적어 주십시오.)
협 조 정 도	1. 상 2. 중 3. 하 *71*
응답 신뢰도	1. 상 2. 중 3. 하 *72*
조사원 이름	지역 / 조사원 ID *73-77*

Supervisor	검 증 원
78-80	

Copyright(c) 2011 Gallup Korea Ltd. All rights reserved. |
