# Supplementary material for: Combination of anxiety and depression is associated with an increased headache frequency in migraineurs: a population-based study
Source: BMC Neurol. 2014 Dec 14;14:238. doi: 10.1186/s12883-014-0238-4 (PMC4279894; doi:10.1186/s12883-014-0238-4)
Supplement: Additional file 4: — Questionnaire-English (http://www.biomedcentral.com/content/supplementary/s12883-014-0238-4-s4.pdf). [file 12883_2014_238_MOESM4_ESM.pdf]

Basic personal information

Residential area:_____(si)/(gun)

Size of residential area

(1) Large cities (2) Small or middle cities (3) Countryside

3. Gender: (men/ women)

SQ3) How old are you?____years old

When were you born in the year of 19 ____

SQ4) What is your age group?

(1) 19-29 years old (2) 30-39 years old (3) 40-49 years old

(4) 50-59 years old (5) 60 years or more

SQ6) Which of these best describe your work?

(1) Agriculture, fishery or stock breeding (2) Self-employed

(3) Sales (4) Skilled worker (5) Skilled worker

(6) Office worker (7) Administrative worker

(8) Professional (9) Housewife (10) Student

(11) Unemployed (12) Retired (13) Other

19. In the past year, have you had at least one headache lasting more than 1 minute?

(Yes)/ (No)

These questions about your headache during the previous 1 year (No. 5-25)

20. On average, how long did these headaches last?

( ) second(s), ( ) minute(s), ( ) hour(s), ( ) day(s),

21. How often did you experience such headaches during the last 1 year?

Daily (times), weekly (time), monthly (times), yearly (times),

22. How bad was your headache?

(1) Headache did not disturb usual daily activities (mild).

(2) Headache often disturbed usual daily activities, but I could perform more than half of my daily activities (moderate).

(3) I can't perform my usual daily activities when I suffer these headaches (severe)

23. How severe was your headache? Please indicate a mark on the line which displays most properly about intensity of your headache. (O is no pain state and 10 is worst possible pain state)

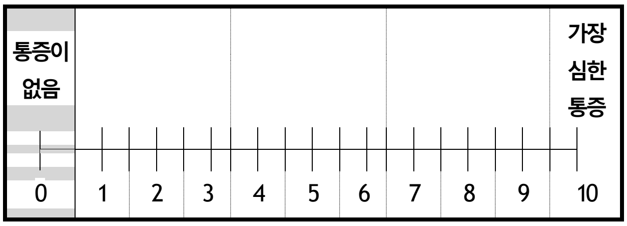

24. What was the location of the headache?

(1) Right side (2) Left side (3) Bilateral side (4) whole head

(5) Unilateral either way (6) Here and there (migrating)

25. Please briefly describe your headache ______________________________

26. What was the headache like? Please statement all describes your headache during the previous year.

(1) Pulsating and throbbing

(2) Heavy and stiff

(3) Tightening feeling like tying a band around your head

(4) Sharp like pinpricking

(5) Sudden and severe like hitting your head with a hammer

(6) Other: describe____________

27. These are questions asking about your headaches.

Question		Yes	No

No.			
(1)	Do you feel sick to your stomach during your headaches?		
(2)	Do you feel nauseated during your headaches?		
(3)	Do you vomit during your headaches?		
(4)	Do light bother you a lot more than when you don't have headaches?		
(5)	The headache worsened by activities such as walking or climbing stairs?		
(6)	Is your headache more painful when you are in noisy surroundings?		
(7)	Do you feel differently or uncomfortable smell sense than you don't have headaches?		
(8)	Do you see scintillating light, glittering stars or experience blurring of vision before or during your headaches?		
(9)	Did you feel dizzy sense before or during your headaches?		
(10)	Did you experience a sudden severe headache?		
(11)	Did you experience unilateral headaches, presenting less than 4 h in a day, for more than 7 days?		
(12)	Did you miss activities in work, school or house shores by headache during the previous 3 months?		
(13)	Did you experience decreased activities in work, school or house shores by headache during the previous 3 months?		

28. These questions asking you the way you feel and what you cannot do because of headache

(1) When you have headaches, how often is the pain severe?				
Never	Rarely	Sometimes	Very often	Always
(2) How often do headaches limit your ability to do usual daily activities including household work, work, school, or social activities?				
Never	Rarely	Sometimes	Very often	Always
(3) When you have a headache, how often do you wish you could lie down?				
Never	Rarely	Sometimes	Very often	Always

(4) In the past 4 weeks, how often have you felt too tired to do work or daily activities because of your headaches?				
Never	Rarely	Sometimes	Very often	Always
(5) In the past 4 weeks, how often have you felt fed up or irritated because of your headaches?				
Never	Rarely	Sometimes	Very often	Always
(6) In the past 4 weeks, how often did headaches limit your ability to concentrate on work or daily activities?				
Never	Rarely	Sometimes	Very often	Always

29. Do you have relatives suffering from headache? Check all

(1) Grandfather (2) Grandmother (3) Father (4) Mother (5) Brother

(6) Sister (7) Siblings (8) Cousin (9) Uncle (10) Aunt

(11) Nephew (12) Other

30. These are questions asking about what you think about you in the last month

Question No.		Yes	No
(1)	Have you felt keyed up, on edge?		
(2)	Have you been worrying a lot?		
(3)	Have you been irritable?		
(4)	Have you had difficulty relaxing?<br>(If “Yes” to tow of the above, go on to 5-9)		
(5)	Have you been sleeping poorly?		
(6)	Have you had headaches or neck aches?		
(7)	Have you had any of the following: trembling, tingling, dizzy spells, sweating, urinary frequency, diarrhea?		
(8)	Have you been worried about your health?		
(9)	Have you had difficulty falling asleep?		

31. On average, when did you go to bed for sleep during the last month?

(1) Weekdays (AM/PM) ____:____

(2) Weekends (AM/OM) ____:____

32. On average, when did you wake up during the last month?

(1) Weekdays (AM/PM) ____:____

(2) Weekends (AM/OM) ____:____

33. On average, when is your sleep duration?

(1) Weekdays __hour__min

(2) Weekend ___hour__min

34. Did you feel that your sleep duration is enough?

(1) Enough

(2) Not enough

35. If your sleep is not enough, what is your expected sleep duration?_____hours___min

36. During the last month, did you take drugs for your sleep? (Yes) (No)

(If you answered “Yes”, go to question 29-1)

37. How often did you take drugs for your sleep?

(1) Less than 1 time per week

(2) 1-2 times per week

(3) More than 3 times per week

38. How do you cope when you are sleepy?

(1) Take coffee or tea (2) Cigarette smoking (3) Exercise

(4) Take a nap (5) Nothing (6) Other

39. When did you go to work or school?

(AM) (PM)______:______

40. Are you shift worker? (Yes) (No)

41. Do you experience accident by sleepiness during the last 3 months? (Yes) (No)

42. These are questions asking about your sleepiness. How likely are you to doze off or fall asleep in the following situations, in contrast to feeling just tired?

0 = would never doze/ 1 = slight chance of dozing

2 = moderate chance of dozing/ 3 = high chance of dozing

Situation	Chance of dozing

Sitting and reading	
Watching television	
Sitting inactive in a public place (eg. theater or meeting	
As a passenger in a car for an hour without a break	
Lying down to rest in the afternoon when circumstances permit	
Sitting and talking to someone	
Sitting quietly after a lunch without alcohol	
In a car, while stopped for a few minutes in the traffic	

43. These questions are asking how often have you had trouble sleeping because you…

	Not during the past month	Less than one a week	Once or twice a week	Three or more times week
Cannot get to sleep within 30 min				
Wake up in the middle of the night or early morning				
Have to get up to use the bathroom				
Cannot breathe comfortably				
Cough or snore loudly				
Feel too cold				
Feel too hot				
Have bad dreams				
Have pain				
Other reason(s), please describe, including how often you have had trouble sleeping because of this reason(s):				

44. How long (in minutes) has it taken you to fall asleep each night? _________min.

45. During the past month, how often have you had trouble staying awake while driving, eating meals, or engaging in social activity?

(1) Not during the past month (2) Less than once a week

(3) Once or twice a week (4) Three or more times week

46. During the past month, how much of a problem has it been for you to keep up enthusiasm to get things done?

(1) Not during the past month (2) Less than once a week

(3) Once or twice a week (4) Three or more times week

47. During the past month, how did you rate your sleep quality

(1) Very good (2) Fairy good

(3) Fairly bad (4) Very bad

48. These questions are asking how often have you been bothered by any of the following problems over the past 2 weeks.

	Not at all	Several days	More than half the days	Nearly every day
Little interest or pleasure in doing things				
Feeling down, depressed or hopeless				
Trouble falling asleep, staying asleep or sleeping too much				
Feeling tired or having little energy				
Poor appetite or overeating				
Feeling bad about yourself-or that you are a failure or have let yourself or your family down				
Trouble concentrating on things, such as reading the newspaper or watching television				
Moving or speaking so slowly that other people could have noticed. Or the opposite-being so figety or restless that you have been moving around a lot more than usual				
Thoughts that you would be better off dead, or of hurting yourself				

49. If you checked off any problems, how difficult have these problems made it for you to do your work, take care of things at home, or get along with other people?

(1) Not difficult at all (2) Some difficult

(3) Very difficult (4) Extreme difficult

50. Do you have insomnia? (Yes) (No)

51. How often did you experience insomnia?

(1) Less than 1 day per week

(2) 1-2 day per week

(3) 3-4 days per week

(4) 5 or more days

52. These are questions asking about your insomnia problems, please rate.

(1) Difficulty falling asleep				
None	Mild	Moderate	Severe	Very severe
(2) Difficulty staying asleep				
None	Mild	Moderate	Severe	Very severe
(3) Problem waking up too early				
None	Mild	Moderate	Severe	Very severe

53. How satisfied/dis satisfied are you with your CURRENT sleep pattern?

(1) Very Satisfied (2) Satisfied (3) Moderately Satisfied

(4) Dissatisfied (5) Very Dissatisfied

54. How noticeable to others do you think your sleep problem is in terms of impairing the quality of your life?

(1) No at all noticeable (2) A Little (3) Somewhat

(4) Much (5) Very Much Noticeable

55. How worried/distressed are you about your current sleep problem?

(1)No at all worried (2) A Little (3) Somewhat

(4) Much (5) Very Much Worried

56. To what extent do you consider your sleep problem to interfere with your daily functioning (e.g. daytime fatigue, mood, ability to function at work/daily chores, concentration, memory, mood, etc.) currently?

(1) No at all interfering (2) A Little (3) Somewhat

(4) Much (5) Very Much interfering

57. Do you have relatives suffering from insomnia? Check all

(1) Grandfather (2) Grandmother (3) Father (4) Mother (5) Brother

(6) Sister (7) Siblings (8) Cousin (9) Uncle (10) Aunt

(11) Nephew (12) Other

58. Do you snore? (Yes) (No) (Don’t know) (If answer no or don’t know, go to No. 63)

59. Your snoring is:

(1) Slightly louder than breathing (2) As loud as talking

(3) Louder than talking (4) Very loud-can be heard in adjacent rooms

60. How often do you snore?

(1) Nearly every day (2) 3-4 times a week

(3) 1-2 times a week (4) 1-2 times a month

(5) Never or nearly never

61. Has your snoring ever bothered other people? (Yes) (No) (Don't know)

62. Has anyone noticed that you quit breathing during your sleep?

(1) Nearly every day (2) 3-4 times a week

(3) 1-2 times a week (4) 1-2 times a month

(5) Never or nearly never

63. How often do you feel tired or fatigued after your sleep?

(1) Nearly every day (2) 3-4 times a week

(3) 1-2 times a week (4) 1-2 times a month

(5) Never or nearly never

64. During your waking time, do you feel tired, fatigued or not up to par?

(1) Nearly every day (2) 3-4 times a week

(3) 1-2 times a week (4) 1-2 times a month

(5) Never or nearly never

65. Have you ever nodded off or fallen asleep while driving a vehicle? (Yes) (No) (If Yes, go to No. 66)

66. How often does this occur?

(1) Nearly every day (2) 3-4 times a week

(3) 1-2 times a week (4) 1-2 times a month

(5) Never or nearly never

67. Do you have, or have you had, recurrent uncomfortable feelings or sensations in your legs while you are sitting or lying down? (Yes) (No)

68. Do you, or have you had, a recurrent need or urge to move your legs while you were sitting or lying down? (Yes) (No)

If you answered YES to either question continue with No. 69

If you answered NO to both questions, go straight to Section B (last page)

69. Are you more likely to have these feelings when you are resting (either sitting or lying down) or when you are physically active? (Resting) (Active)

70. If you get up or move around when you have these feelings do these feelings get any better while you actually keep moving? (Yes) (No) (Don't know)

71. Which times of day are these feelings in your legs most likely to occur?

(Please circle one or more than one)

(Morning) (Mid-day) (Afternoon) (Evening) (Night) (About equal at all times)

72. Will simply changing leg position by itself once without continuing to move usually relieve these feelings?

(1) Usually relieves (2) Does not usually relieve (3) Don't know

73. Are these feelings ever due to muscle cramps? (Yes) (No) (Don't know)

(If No, go to No. 75)

74. If so, are they always due to muscle cramps? (Yes) (No) (Don't know)

75. Do these feelings occur only when sitting or only when lying down?

(1) Neither (2) Only when sitting (3) Only when lying down

(4) Both when sitting and when lying down

76. When you actually experience the feelings in your legs, how distressing are they?

(1) Not at all distressing (2) A little bit

(3) Moderately (4) Extremely distressing

77. In the past 12 months, how often did you experience these feelings in your legs?

(please circle only one answer)

(1) Every day (2) 4-5 days per week (3) 2-3 days per week

(4) 1 day per week (5) 2 days per month (6) 1 day per month or less

(7) Never

78. Approximately how old were you when you first noticed these feelings in your legs? (please write age) _____years

79. Do you have relatives suffering from similar symptoms mention at No.67-No. 78? Check all

(1) Grandfather (2) Grandmother (3) Father (4) Mother (5) Brother

(6) Sister (7) Siblings (8) Cousin (9) Uncle (10) Aunt

(11) Nephew (12) Other

80. How often do you alcohol drink?

(1) Nearly every days (2) 4-6 days per week (3) 2-3 days per week

(4) 1day per week (5) 2-3 days per month (6) 1 day per month

(7) Quit drinking (8) Never drink

81. Do you smoke?

(1) Currently smoking (2) Quit smoking (3) Never smoke

82. How often do you exercise more than moderate intensity?

(1) Nearly every days (2) 5-6 days per week

(3) 3-4 days per week (4) 1-2 days per week

83. Please describe your current state of diseases mentioned as below

	Yes	No	Onset age (Age)
Stoke			
Myocardial infarction			
Peripheral vascular disease			
Angina			
Hypertension			
Diabetes Mellitus			
Hyperlipidemia			
Other cardiac disease			

84. Do you have other diseases listed in No. 83? If so, please describe.

(1)_______________________________

(2)_______________________________

(3)_______________________________

85. What is your weight?________Kg

What is your height?________cm

D-6. How much schooling have you had?

(1) Elementary school graduated or less

(2) Middle school graduated

(3) High school graduated

(4) College graduated or college student

(5) Graduate school graduated or Graduate school student

D12-2. What is your family's approximate monthly income ?

(1) Less than 490,000 KRW (Korean won)

(2) 500,000-990,000 KRW

(3) 1,000,000-1,490,000 KRW

(4) 1,500,000-1,990,000 KRW

(5) 2,000,000-2,490,000 KRW

(6) 2,500,000-2,990,000 KRW

(7) 3,000,000-3,490,000 KRW

(8) 3,500,000-3,990,000 KRW

(9) 4,000,000-4,490,000 KRW

(10) 5,000,000-5,990,000 KRW

(11) 6,000,000-6,990,000 KRW

(12) 7,000,000-7,990,000 KRW

(13) 8,000,000-8,990,000 KRW

(14) 9,000,000-9,990,000 KRW

(15) More than 10,000,000 KRW

(16) No income
